# A Manual of Natural Therapy

**Published:** 1910-03

**Authors:** 


**A Manual of Natural Therapy.** By THOMAS D. LUKE, M.D., Pp. xiv, 302. Bristol: John Wright & Sons Ltd. 1908.

To very many, if not to a majority, of medical men a hydro is an establishment consisting of an hotel where there are provided Turkish baths, sitz baths, air douches, Fango treatment, hydro-electric baths, Dowsing baths and the like, and so on, the names in some cases being the extent of the medical man's knowledge of them.

The aim of this book is to present an outline of the methods of hydropathic, electrical and electro-thermal treatment, the physiological actions and the indications and contra-indications in disease. Such a volume has long been wanted to fill a gap on our reference bookshelves. It will save the practitioner some wriggling when he is asked by patients the relative values of, say, the sinusoidal and the high-frequency currents, or of the Dowsing and Fango treatments. The medical man has had little opportunity of personally observing these methods, and the consequent haziness about them has done much to encourage quackery.

The Kneipp Kur is here dismissed in four or five lines as a form of quackery, and since the author has held office in a hydro which bears the name of Smedley, he will not object to its being pointed out that there are several doctors (Baumgarten, Schultz and others) in Wörishofen who control baths and regulate the Kur on scientific and strictly professional lines; indeed, the Kneipp method of regulating the various cold douches according to the individual is more scientific than the methods given in Dr. Luke's book.

The title of this book, *Manual of Natural Therapy*, is not justified by the sub-title, "Hydrotherapy, Massage, Electricity, Diet." Under such title Climate and Health Resorts, Swedish and other Curative Exercises, and Lavage, amongst others, should have been included. It would be better to leave out the subjects of diet and mineral waters (the latter being represented in this book by the foreign varieties only), and further enlarge the sections on massage, hydropathic and electrical treatments, upon which Dr. Luke shows himself well qualified to speak with authority, and the usefulness of the book would be much increased.

The free use of excellent illustrations adds greatly to the value of the book, and the form in which it has been published is fully up to the high standard to which Messrs. John Wright have accustomed us.

